# Spatial Analysis of Cultivated Land Productivity, Site Condition and Cultivated Land Health at County Scale

**DOI:** 10.3390/ijerph191912266

**Published:** 2022-09-27

**Authors:** Fengqiang Wu, Caijian Mo, Xiaojun Dai, Hongmei Li

**Affiliations:** 1 School of Environment and Resource, Southwest University of Science and Technology, 59 Qinglong Road, Mianyang 621010, China; 2 Tianfu Institute of Research and Innovation, Southwest University of Science and Technology, Zizhou Avenue Road, Chengdu 610213, China; 3 Mianyang S&T City Division, National Remote Sensing Center of China, 125 Biyun Road, Mianyang 621002, China; 4 School of Civil Engineering and Geomatics, Southwest Petroleum University, 8 Xindu Road, Chengdu 610500, China; 5 Mianyang Natural Resources Bureau and Municipal Planning, No. 2, Yunquan South Street, Mianyang 621000, China

**Keywords:** cultivated land productivity, site assessment, health assessment, coupling coordination relationship, sustainable development

## Abstract

Cultivated land is a fundamental factor related to the social stability and sustainable development of the whole country. However, the safety of quantity and quality of cultivated land has decreased year by year, resulting in great challenges to the sustainable development of cultivated land. Cultivated land productivity, site conditions, and soil health jointly determine the sustainable development potential of cultivated land. Analyzing and calculating the coupling and cooperative relationship between these three subsystems can provide a theoretical and methodological reference for protecting and zoning cultivated land resources. Using Jiangyou City as a case study, this paper constructs a coupling coordination degree model of cultivated land productivity, site conditions, and soil health assessment systems in different geomorphic regions, and comprehensively analyzes the level of sustainable development of cultivated land in the study area. The results show that there are differences in the development potential of cultivated land resources in the mountainous regions in the north, the hilly regions in the center, and the plain regions in the south of Jiangyou City. The coupling coordination index of the three regions were calculated as 0.34, 0.51, and 0.63, respectively, for which the overall average coupling coordination index is 0.57; notably, it only reaches the “barely coordination” level. Based on our analysis results, the cultivated lands in Jiangyou City are classified into the following zones: core protection zone, dominant remediation zone, and key regulation zone. The cultivated land located in the core protection zone has a high coupling coordination index, which can be used as the preferred area for the delimitation of high standard basic farmland and permanent basic farmland. For the cultivated land located in the dominant remediation zone, the development of its subsystems is unbalanced. Comprehensive land improvement projects can be carried out in this zone to improve the overall quality. For the cultivated land located in the key regulation zone, it is recommended to implement projects such as returning farmland to forests to improve land use efficiency. In particular, the evaluation index system constructed in this paper is sufficiently representative, as it can support the classification, quality improvement, and sustainable use of cultivated land. Thus, other similar countries and regions can learn from the evaluation system constructed in this paper.

## 1. Introduction

Food security is intrinsically related to national stability. Cultivated land is a key national land resource and the most important strategic resource for ensuring national food security. Today, population pressures, wars, and associated land-use changes continuously increase the burden on global soil resources. At the same time, soil degradation has increased, affecting food security and sustainable agricultural development [1,2,3]. Therefore, rational use of land and protection of cultivated land are important prerequisites for sustainable agricultural development [4]. At present, the state requires the overall protection of cultivated land from the perspective of quantity, quality, and ecology. However, the loose management mode cannot support the needs of current farmland protection and management; therefore, it is urgent to build a three-dimensional farmland quality evaluation system to meet the needs of current farmland quality evaluation and protection management [5,6]. Many scholars have deepened their understanding of cultivated land quality, mainly studying the connotation of cultivated land quality from the aspects of soil quality, ecological quality, environmental quality, management quality, economic quality, aesthetic, and cultural quality [7,8,9,10]. From the perspective of cultivated land resources, cultivated land system is a natural and social complex composed of productivity, site conditions, and soil environment. The natural factors of cultivated land, including climate, landform, and soil, are relatively stable and difficult to change in a short time. The site conditions are determined by human activities, mainly including farmland infrastructure condition, labor input, machinery input, and other factors. It is relatively variable and will continue to improve. The productivity, site conditions, and health of cultivated land jointly determine its sustainable development potential [11]. To fully grasp the internal and external attributes of cultivated land, we used a spatial analysis method to study the degree of coupling and coordination between cultivated land productivity, site conditions, and health, so as to provide assistance for cultivated land management and planning. 

The theory, technical methods, and model research of cultivated land quality protection are constantly improving to protect stable, high-yield cultivated land resources. In particular, the cultivated land productivity assessment (PA) is constantly expanding. Now, its evaluation indicators cover a range of topics, including natural quality, utilization conditions, spatial-temporal distribution, and farmers’ behavior [12,13,14,15,16,17], and the selection of these evaluation factors is based on objective, mathematical methods. For example, the geographic detector and Delphi methods are often used to construct the evaluation systems of cultivated land productivity [16,18], while the ecological risk model, agro-ecological zones model, crop growth model, and others are used to evaluate the productivity of cultivated land [19]. With the development of technology, regional cultivated land productivity is evaluated by means of remote sensing, such as using normalized difference vegetation index (NDVI), net primary productivity (NPP), and other factors calculated by remote sensing [20].

Cultivated land site conditions are the primary indicators for guaranteeing the stable and sustainable use of cultivated land resources. The cultivated land site assessment (SA) mainly consists of the assessment of the surrounding, locational, and ecological conditions of the cultivated land in question. The SA system is often based on comprehensive tools and multi-objective methods, such as GIS and remote sensing technology [21,22,23,24]. Through this evaluation model, the SA system is constructed based on different landform types [25]. Mathematical statistical modeling is key in many SA systems, such as the LESA model [26,27].

Cultivated land is one of the most important agricultural resources. However, the excessive application of chemical fertilizers and high-intensity utilization have had significant effects its health. The resulting shallow arable layer, significant decrease in organic matter in the surface soil, and risk of exceeding the standard of heavy metals in the soil have not only aggravated the deterioration of the ecological environment of cultivated land, but have also reduced grain production and caused a decline in the quality of agricultural products. These issues have attracted extensive attention in recent years. The significance of cultivated land health is rich and diverse and cannot be well understated [28,29,30]. The cultivated land health assessment (HA) mostly focuses on agricultural ecosystem and soil health [31,32,33]. Moreover, the index of the HA is often selected as one of the indexes of the evaluation of cultivated land quality [34,35]. From the analysis of evaluation factors, the HA of cultivated land selects indicators from the aspects of cultivated land quality, productivity, and environment [36,37].

However, while most of the above studies focus on constructing the cultivated land assessment layer, they lack any analysis which considers the synergy and relationships between the three subsystems of cultivated land: PA, SA, and HA. Based on the land evaluation and site assessment (LESA) theory [38], this study takes the different geomorphic areas of Jiangyou City as research units and constructs the PA, SA, and HA systems of these units of cultivated land. The cultivated land’s PA system is assessed through natural factors, such as soil and geographical factors, the SA system is assessed through the cultivated land’s utilization factors and surrounding factors, whereas the HA system is assessed through an internal factor (soil heavy metal pollution) and an external factor (fractional vegetation coverage). The spatial characteristics of cultivated land productivity, site conditions, and cultivated land health in different geomorphic areas are studied through the cooperative relationship between two or three systems. Based on the analysis results of coupling coordination degree, the protection level of cultivated land in the study area is determined. Ultimately, this study provides a theoretical and methodological basis for the permanent protection of arable land resources.

## 2. Material and Methods

### 2.1. Study Area

**Figure 1:**
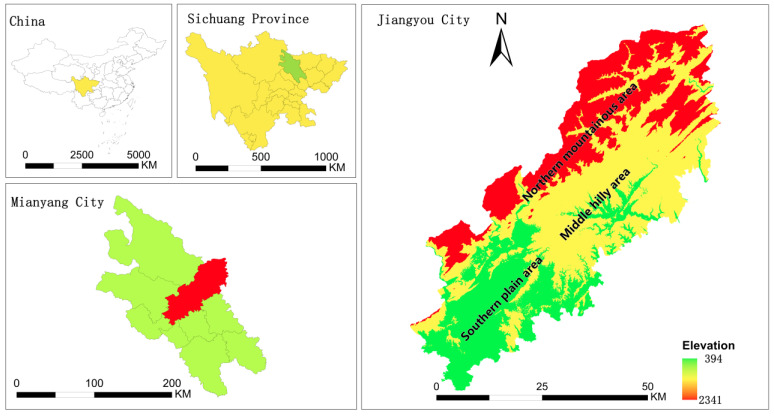
Location and topography of Jiangyou city.

Jiangyou City is located in Mianyang City (Figure 1), Sichuan Province, on the northwest edge of Sichuan Basin and in the north of the Longmen Mountains regions. Its elevation is between 300 and 2400 m. The terrain in the territory is high in the north and low in the south. Notably, this area was severely affected by the Wenchuan earthquake in 2008.

The data used in this study includes: (1) “Jiangyou City Land Use Status Database”, which is used to extract the basic land type information such as cultivated land, slope, urban center, water system, and traffic roads; (2) the soil properties of cultivated land as extracted from the “Jiangyou City Cultivated Land Quality Grade Update Database”, including soil pH, soil organic matter content, available soil depth, etc.; (3) Landsat 8, which is used to calculate the fractional vegetation cover (FVC) index of the study area; (4) the “Jiangyou City Soil Database”, which we used to extract the content information of heavy metals in the soil of the study area from a total of 308 sample points, including: cadmium, mercury, arsenic, lead and chromium. The spatial distribution map of heavy metals in the region was interpolated from these points; (5) the “Jiangyou Yearbook 2019”, which was used to obtain population data. The data sources are shown in Table 1.

### 2.2. Research Framework

The basic function of cultivated land is production, which can directly meet human survival needs and help maintain agricultural sustainable development. The production function is the ability of cultivated land resources as an ecosystem to maintain the stability of the human and ecological environment. It is the function of exchanging material, energy, and information between the internal resources of cultivated lands and the external natural environment. Good site conditions are necessary for the sustainable and healthy development of cultivated land, as it ensures sustainable production [26]. The soil health conditions of cultivated land play an integral role in maintaining its productivity, improving environmental quality, and promoting the healthy growth of plants. Healthy soil has good structure, function, and buffering performance, and can continuously maintain its structure and function, as well as the dynamic balance of the soil ecosystem [39].

In the cultivated land resource system, each subsystem does not exist and develop in isolation; instead, a coupling relationship exists between the two of them. For example, there is a coupling effect between “soil properties” in the PA subsystem and FVC in the HA subsystem [40]. These elements constitute an orderly structure of cultivated land resources, which is the condition for maintaining their integrity. Therefore, in order to maintain the unified management of natural resources, it is necessary to integrate the existing cultivated land evaluation system and establish a scientific, comprehensive, and unified arable land protection evaluation system.

**Figure 2:**
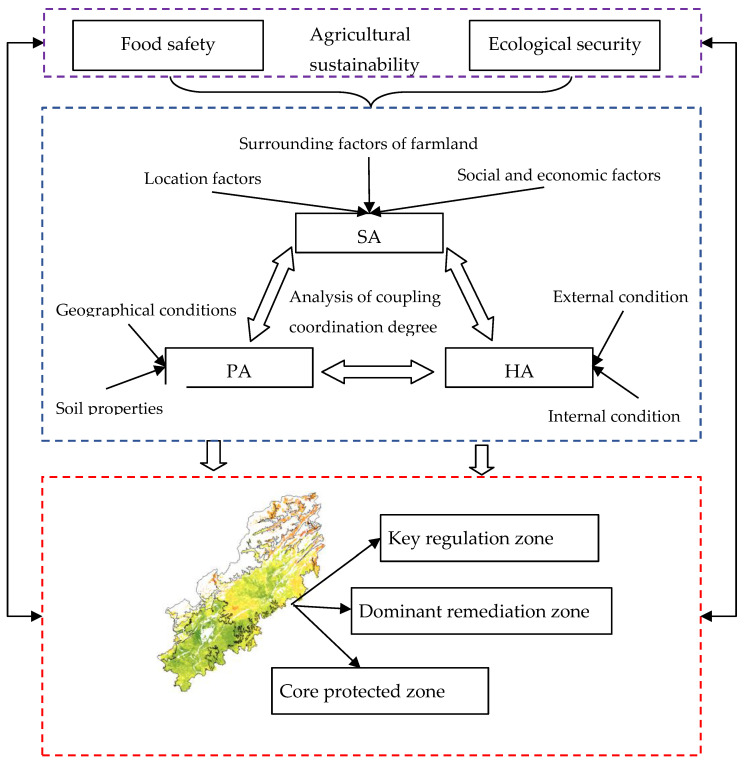
The coupling coordination relationship flow chart of PA, SA, and HA.

In order to integrate the existing evaluation system and the proposed innovation path, we have established a new evaluation framework, as seen in Figure 2. The framework is composed of three independent evaluation systems based on human needs: PA, SA, and HA. The PA system focuses on the assessment of cultivated land’s production capacity. The SA subsystem is used to assess the ability of guaranteed conditions for the sustainable development of cultivated land. As cultivated land soil health is closely related to cultivated land quality, the HA subsystem is used to assess the quality of the cultivated land soil. Cultivated land units with different functions delineated through the coupling between the subsystems, thus reflecting different service functions. According to the analysis of the coupling results of the three subsystems of cultivated land, cultivated land as used here is divided into three zones: the key regulation zone, dominant remediation zone, and core protected zone. The results of this analysis can provide a basis for the classified protection of cultivated land and the division of permanent basic farmland.

### 2.3. System of Cultivated Land PA, SA, and HA

The study takes a sample of 48,693 cultivated land parcels from Jiangyou City in 2019 as the evaluation unit. First, the projection transformation and vectorization of each factor were carried out using ArcGIS software. Second, the scores of each parcel were calculated based on the results of the first step, as shown in Table 2. Factors such as land surface slope, available soil depth, etc., are graded according to national arable land standards or related research [25,41]. Soil heavy metal is classified according to national standards [42]. Other factors such as farmland road density, fractional vegetation cover, etc., are graded according to the principle of equal spacing. Third, the scores of each cultivated land unite in the PA, HA, and SA systems were normalized [43]. Fourth, the four types of coupling coordination index values of each parcel were calculated [44,45]. The scores of each evaluation factor are as follows:

#### 2.3.1. PA System

In recent years, the results of updating and improving the agricultural land quality classification have provided a rich and detailed theoretical and practical foundation for the establishment of the cultivated land PA system. For this study, the PA system of cultivated land was constructed according to the evaluation system of agricultural land classification (Table 2) [46]. Then, land surface slope, available soil depth, soil texture, soil organic matter, profile pattern, and soil pH were selected for the PA system.

The available soil depth is closely related to the soil quality of the cultivated land. Within a certain range, the thicker the soil layer, the more fertile the soil. This is because soil texture has a significant effect on soil water and heat conditions, fertility conditions, and root development. Notably, the quality of soil texture can be reflected in the soil’s clay content [28]. The degree of acidity and alkalinity is also an important factor in soil quality, as the normal growth of crops can be restricted by excessive acid or alkali content in the soil. This is expressed as a pH value, among which neutral soil is the most fertile. Meanwhile, the content of soil organic matter is positively correlated with soil fertility, soil structure, and soil buffer capacity. The index of each parcel unit is calculated by weighted superposition, after which the final score of the PA is expressed using the normalization method.

In this paper, the cultivated land patch is taken as the evaluation unit, from which the cultivated land productivity index map is created using the weighted superposition of the above eight factors. The calculation formula is shown in Formula (1):

(1) $$F_{i}=\overset{n}{\underset{i=1}{\sum }}\left(f_{ij}\cdot \omega_{ij}\right)$$

where: $F_{i}$ is the score of the ith parcel;$\;f_{ij\;}$ is the score of the jth factor of the  ith unit; $\omega_{ij}$ is the weight of the jth factor of the ith parcel, and n is the number of factors.

#### 2.3.2. SA System

Cultivated land site conditions indicate a location’s potential for the stable and sustainable use of its cultivated land resources [47]. The strength of a site’s elements indicates the development potential of cultivated land resources and can be measured using he SA subsystem. This is evaluated using three indicators: location factors, farmland capital construction, and social and economic factors. The distance of a plot of land from the city center corresponds to the willingness of farmers to develop their cultivated land. The closer the land is to the city, the higher the farmers’ desire to develop. At the same time, a plot of land’s distance from the farmers’ market indicates how convenient it is for farmers to buy seeds, fertilizers, and tools. The patch shape index indicates the intensity with which cultivated land can be developed [48]. The more regular the cultivated land, the higher the degree to which it can be developed. The water network density indicates the ease with which the farmland can be irrigated. The higher the index, the easier it is to irrigate. The density index of the farmland road network indicates the convenience of reaching the cultivated land and if it is possible to sow and harvest the cultivated land mechanically. The per capita cultivated land represents the pressure of the population on the cultivated land. In the SA subsystem, each factor is first calculated individually, then the cultivated land SA index of the entire study area is calculated and analyzed using the weighted superposition method.

(1) Calculation of the point factor

The degree of urban influence and degree of the agricultural market influence are calculated by a point-like influence formula. The point-like influence mode is expressed by concentric circle diffusion and is calculated using the linear attenuation method to express the score of point factors [49], as shown in Formula (2):

(2) $$f_{i}=M_{i}\left(1-r_{i}\right),\left(r_{i}=\frac{d_{i}}{d}\right)\;\;\;\;$$

where: $f_{i}$ is the score of point factors on the evaluation unit at a certain relative distance; $M_{i}$ is the scale index; $d_{i}\;$ is the absolute distance from the evaluation unit to the factor; d is the influence radius of the factors; $r_{i}$ is the relative distance of the factor.

(2) Patch shape index

The patch shape index is comprehensively expressed by patch area and perimeter. The calculation formula is written as follows [50]:

(3) $$f_{i}=\frac{\sqrt[{4}]{S_{i}}}{P_{i}}$$

where:$\;f_{i}$ is the i th patch shape index; $S_{i}$ is the patch area (m^2^); and $P_{i}$ is the perimeter of the patch (m). The closer the $f_{i}$ value is to 1, the closer the cultivated land patch shape is to a banded quadrilateral, which is easy to cultivate. At the same time, the more irregular the patch shape, the more difficult it is to use.

(3) Water network density index

The index of the water network density is the ratio of the total area of rivers and lakes to the total area of the assessed area. This represents the abundance of water resources. The calculation formula is shown in Formula (4) below:

(4) $$f_{i}=A_{riv}\times \frac{s_{riv}}{S}+A_{lak}\times \frac{s_{lak}}{S}\;\;\;\;\;$$

where: $f_{i}$ is the ith water network density in village; $A_{riv}$ is the normalized index of river length, $\;A_{lak}$ is the normalized index of lake area, $\;s_{riv}$ is the area of the river, $s_{lak}$ is the area of the lake, S is the area of arable land in village.

(4) Farmland road density

The index of farmland road density is calculated using the ratio of the length of the farmland road network to the assessed area. This represents the abundance of roads in the region. The greater the index value, the more convenient it is to reach cultivated land. The calculation formula is shown in Formula (5):

(5) $$f_{i}=\sum \frac{L_{i}}{S_{i}}\;\;\;$$

where: $f_{i}$ is the index of cultivated land road density in village; $S_{i}$ is the area of arable land in village; $L_{i}\;$ is the total length of the farmland road in village.

#### 2.3.3. HA System

Healthy cultivated soil can guarantee healthy agricultural production and maintain the multiple functions of the soil ecosystem. In this paper, the external characterization factor of cultivated land soil(FVC) and the internal factor (heavy metal pollution) were selected to evaluate the health of the cultivated land’s soil [51]. The index of the FVC indicates the growth of crops on cultivated land to a certain level. Beyond this, however, certain harmful human activities or other natural factors may lead to the enrichment of heavy metals in the soil, which ultimately threatens the soil quality, the safety of agricultural products, and human health. In this paper, five specific indicators were selected to evaluate the pollution degree of soil heavy metals: cadmium, mercury, arsenic, lead, and chromium.

The HA index is calculated by weighting the sum of Internal HA and External HA. The calculation formula is shown in Formula (6):

(6) $$HCI=A_{int}\times P+A_{ext}\times F$$

where:  HCI represents the index of cultivated land health,  P is the index of soil heavy metal pollution,  F is the fractional vegetation coverage, $A_{int}$ and $A_{ext}\;$ are the weights of P and  F. The weight is calculated by entropy weight method (EWM) [52].

In this study, the index of soil heavy metal pollution was evaluated using the Nemerow pollution index. First, based on the spatial characteristics of the study area, we selected the five aforementioned heavy metal indicators of cadmium, mercury, arsenic, lead, and chromium for testing, with a total of 325 sampling points. Second, through the interpolation method, the heavy metal pollution index of the whole area was generated and the internal health index of cultivated land was calculated according to the Nemerow comprehensive pollution index method [53]. The Nemerow pollution index P was calculated as shown in Formulas (7) and (8).

(7) $$P=\sqrt{\frac{{\left(\frac{1}{n}\sum P_{i}\right)}^{2}+P_{imax}^{2}}{2}}$$

(8) $$P_{i}=\frac{D_{s}^{i}}{D_{n}^{i}}\;\;\;\;$$

where: P is the Nemero pollution index, $P_{imax\;}$ is the maximum value of pollution index of heavy metal ith  factor. $P_{i}\;$ is the ith single factor pollution index of heavy metals in soil, $D_{s}^{i}$ the measured value of heavy metals in soil, and $D_{n}^{i}$ is the standard value of heavy metals in soil.

For external health status, FVC is used to represent the external health characteristics of cultivated land [54]. The calculation of this index is shown in Formula (9):

(9) $$F=\frac{NDVI-NDVI_{min}}{NDVI_{max}-NDVI_{min}}$$

where: F refers to the index of FVC; $\;NDVI_{min}$ is the value when there is bare soil or no vegetation cover, and $\;NDVI_{max}$ is the value of high vegetation coverage; NDVI is the normalized vegetation index.

### 2.4. Methods

With the in-depth understanding of system theory and healthy development, the coupling coordination degree model has become an important index for representing the interaction between multiple systems in this research field of land productivity. Cultivated land is maintained via a complex system, and there are internal coupling relationships that connect its production, social security, and ecological functions [45,55]. Therefore, the coupled and coordinated evaluation of the multi-system of cultivated land can facilitate the recognition of information on cultivated land resources from the overall development level. In this paper, the coupling coordination degree model is used to study the coupling coordination relationship among cultivated land multi systems. 

According to the coupling coordination degree theory [56,57,58,59], the coupling coordination degree value (D) ranges from 0 to 1. The closer it is to 1, the more harmonious the coupling relationship between these subsystems. In this paper, the coupling coordination relationship indexes of the PA-SA, PA-HA, SA-HA, and PA-SA-HA are calculated using Formula (12). The calculation formula for the coupling coordination relationship is as follows:

(10) $$C=\sqrt[{2}]{\frac{U_{1}U_{2}}{{\left(\frac{U_{1}+U_{2}}{2}\right)}^{2}}}\;\;or\;C=\sqrt[{3}]{\frac{U_{1}U_{2}U_{3}}{{\left(\frac{U_{1}+U_{2}+U_{3}}{3}\right)}^{3}}}\;\;\;\;\;\;\;\;\;\;\;\;\;\;\;$$

(11) $$\;\;\;\;T=W_{1}U_{1}+W_{2}U_{2},W_{1}+W_{2}=1\;\;\;\;\;or\;T=W_{1}U_{1}+W_{2}U_{2}+W_{3}U_{3},W_{1}+W_{2}+W_{3}=1\;$$

(12) $$D=\sqrt{C\cdot T}$$

where: D is the coupling coordination degree index between systems, and C is the coupling degree index between systems; $\;U_{1},U_{2},U_{3}$ are the cultivated land PA, SA and HA index. $W_{1},W_{2},W_{3}$ is the weight index of each subsystem. The weight is calculated by EWM.

## 3. Results and Discussion

### 3.1. Analysis on the Coupling Coordination Relationship of Different Geomorphic Regions

The study area used in this paper is divided into three areas according to its topographic and geomorphic characteristics: southern plain area (SPA), middle hill area (MHA), and northern mountain area (NMA). There are 24,535 cultivated land parcels in SPA accounting for a total area of 380.85 km^2^, 23,056 cultivated land parcels in MHA accounting for a total area of 424.01 km^2^, and 1083 cultivated land parcels in NMA accounting for a total land area of 24.98 km^2^. The coupling coordination index between PA-SA, PA-HA, SA-HA, and PA-SA-HA in different geomorphic areas were calculated and analyzed, the results are shown in the following figure:

To compare and analyze the differences in coupling coordination among cultivated land systems in different geomorphic areas, we took the calculation results of the coupling coordination degree as the evaluation unit, then clustered the scores of all evaluation units according to the K-means clustering method. Five cluster centers were determined for each type of region [60]. The K-means clustering method can analyze the internal structure of a large amount of data. The differences and internal characteristics of the coupling coordination of different geomorphic areas can be found through the horizontal comparative analysis of the clustering centers and the number of clustering cases in different geomorphic areas.

#### 3.1.1. Coupling Coordination Relationship and Spatial Analysis of PA-HA

**Figure 3:**
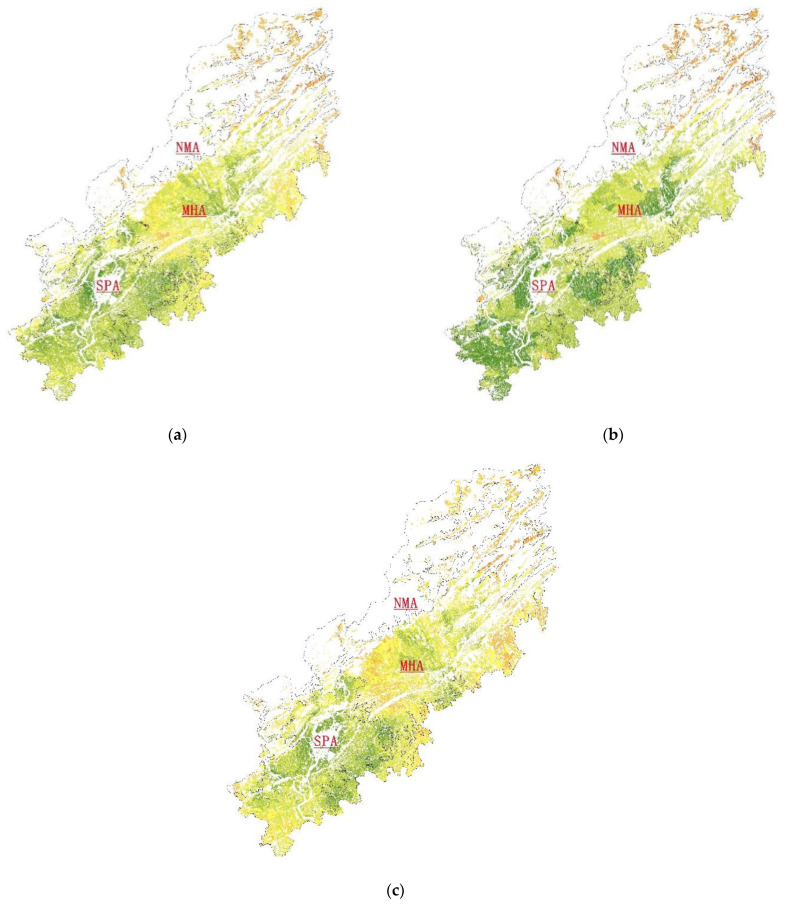

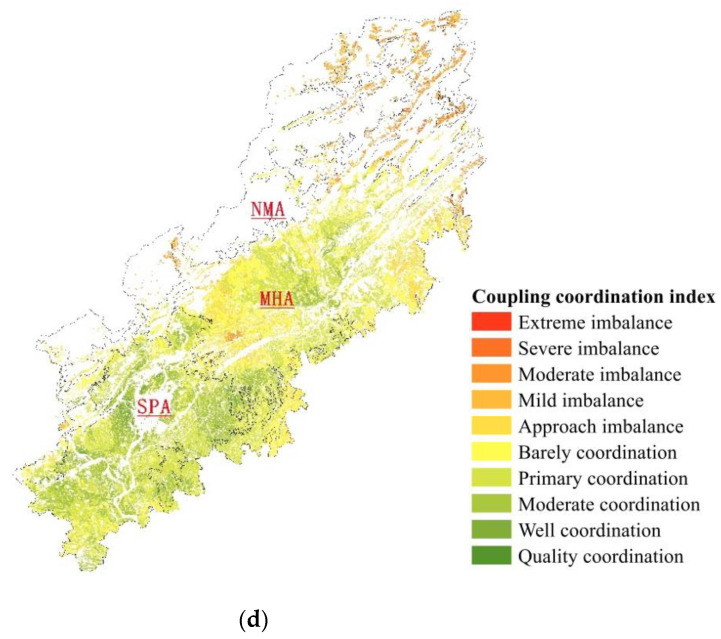
The map of the coupling coordination index of PA-SA (**a**), PA-HA (**b**), SA-HA (**c**) and PA-SA-HA (**d**).

**Figure 4:**
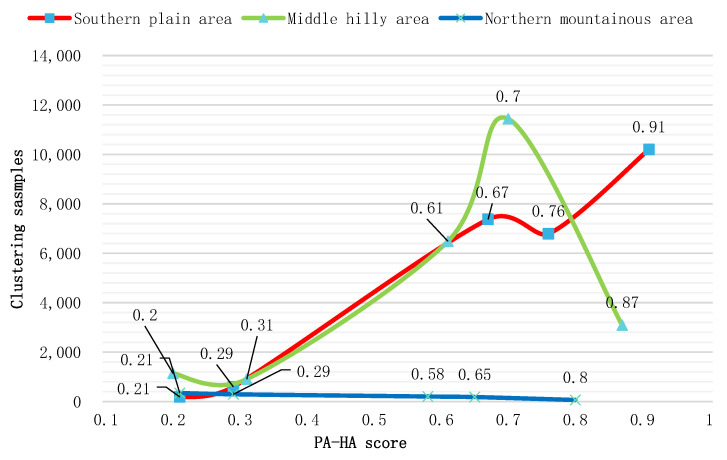
Clustering scores of the coupling coordination index of PA-HA.

The coupling coordination indexes of PA-HA in different geomorphic regions were calculated separately (Figure 3a), the results of which revealed an overall coupling coordination index of 0.71. In the NMA area, the mean index of the PA-HA coupling coordination degree was 0.29, indicating that cultivated land productivity and cultivated land health are in a moderate imbalance level. In the MHA, the mean index of the PA-HA coupling coordination degree was 0.68, indicating that the PA and HA are in the primary coordination level in the area. In the SPA region, the mean index of PA-HA coupling coordination degree was 0.78. This indicates a moderate coordination level, which is higher than that of the other regional systems. These results show that the cultivated land’s productivity and health are in good condition. According to the comparative analysis of the K-means clustering method (Figure 4), the distribution and peak order of the PA and HA coupling coordination indexes are SPA > MHA > NMA, as the cultivated land productivity and cultivated land health in the plain area is higher. In hilly and mountainous areas, although the HA index is high, the PA index is low due to the influence of terrain slope and soil organic matter.

Based on our quantitative analysis, the number of parcels with coupling coordination index greater than 0.5 in the MHA was 21011, accounting for 91% of the total cultivated land in the MHA. In the SPA, the coupling coordination index and the number of cultivated land patches tended to increase linearly. The number of parcels that were at the level of “barely coordination” or better in the SPA accounts for 96% of the area examined. Notably, the number of parcels at the “quality coordination” level exceeds 10,000. That is to say, the majority of parcels had a good coordination between PA and SA. Although the coupling index of the NMA also had a large value (0.8), the number of farmland parcels was small. The overall analysis shows that the northern part of the study area had poor coordination, while the southern part had generally higher coupling, as the cultivated layer in this region is thicker and the soil texture of the cultivated layer is mainly loamy, with a few silty loam and clay loam areas. These factors ensure that the farmland can be productive regardless of drought or flood.

#### 3.1.2. Coupling Coordination Relationship and Spatial Analysis of PA-SA

**Figure 5:**
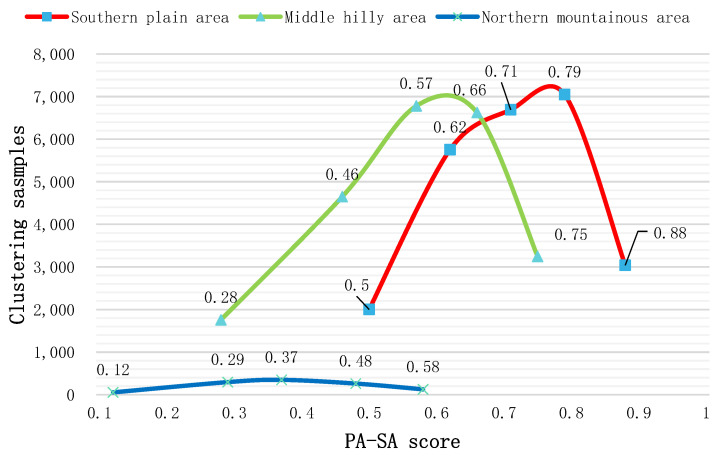
Clustering scores of the coupling coordination index of PA-SA.

This study calculated the coupling coordination index of PA-SA under different geomorphic conditions (Figure 5), the results of which revealed that the overall coupling coordination index was 0.64. In the NMA, the mean index of the PA-SA coupling coordination degree was 0.37, while the maximum was only 0.62, indicating “mild imbalance” level. According to the results, only 125 parcels in this area are considered above the “barely coordination” level. Even so, the index distribution range of PA-SA of cultivated land in the NMA is large, indicating that there were large coordination differences between cultivated land parcels. Compared with other regions, however, this is a low level. As shown in the change trend diagram of the coupling coordination index of the SPA and MHA, the mean index value of the SPA was 0.72, while the MHA was 0.58. For the cultivated land distributed in these two regions, the coupling coordination index of PA-SA shows a normal distribution trend. The index indicates that in these areas, the cultivated land productivity and site conditions are relatively coordinated and have mutual promotion [61]. In the MHA, the number of parcels with a coupling coordination index of less than 0.5 is 6404, only accounting for 27% of the total number in the area. Almost all the cultivated land in the SPA is above the “barely coordination” level.

Figure 3b shows that the cultivated land in the SPA and MHA is closer to the city center and possesses developed road networks and sufficient irrigation. This makes farmers more willing to invest in and improve the quality of cultivated land, which can be accomplished through increasing the nitrogen, phosphorus, and potassium content of the soil through fertilization. The data also demonstrate that the cultivated land in the SPA and MHA could achieve better, more stable development in terms of productivity and site conditions. This mode is a dynamic and coordinated process, which can ensure the friendly development of the cultivated land system under the condition that one side is weak in development and the other side plays a leading role. In the early stage, the cultivated land site conditions are poor, the enthusiasm of farmers is not high, and the productivity of cultivated land is low. In the later stage, as the site conditions of cultivated land become better, such as new roads, farmers or the government are willing to improve the quality of cultivated land, such as implementing land consolidation and development projects. On the other hand, in areas with high-level cultivated land productivity, in order to improve farmers’ willingness to work, the government has made great efforts to improve the site conditions of cultivated land, such as investing in new water conservancy facilities and road networks, so as to maintain a healthy development between productivity and the site conditions of cultivated land.

#### 3.1.3. Coupling Coordination Relationship and Spatial Analysis about SA-HA

After calculating the coupling coordination index of the SA-HA of the cultivated land in the SPA, MHA, and NMA, respectively (Figure 3c), we found the overall coupling coordination index of 0.59. In the NMA, the distribution range of the coupling coordination index was (0.18–0.71), and the mean value was 0.39, which is considered as being at the level of “mild imbalance”. In this area, there were only 40 parcels with a coupling coordination index greater than 0.5. In the MHA, the distribution range of coupling coordination index was (0.22–0.96), with an average value of 0.53. In this region, the number of parcels below the “barely coordination” level was 7871, accounting for 34% of the total. Analyzing the area with low coupling coordination index, we found that although the SA index of this area was high, the HA index was low due to its close proximity to cities and frequent human activities. 

**Figure 6:**
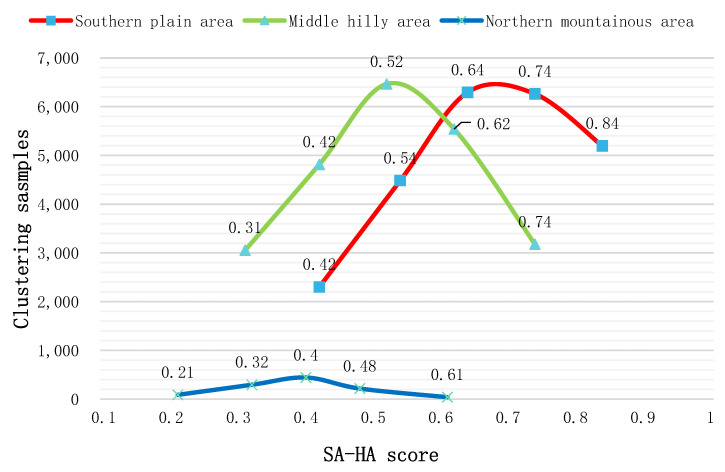
Clustering scores of the coupling coordination index of SA-HA.

According to the curve seen in Figure 6, in the SPA, the five cluster center values were calculated as: 0.42, 0.54, 0.64, 0.74, and 0.84, whereas the average coupling coordination index was 0.67. Of this, 90% of the cultivated land is above the coordination level. Notably, 11,457 parcels are above the “moderate coordination” level, thus accounting for 47% of the total. The region is flat and close to the urban center with a developed road network and water system. Here, the government has strict control over land, so the coupling coordination degree was high in our analysis. Overall analysis shows that although the SA and HA systems can achieve coordinated and interactive development in some regions, the coordination relationship in most regions is weak. This is particularly true for the NMA, where although the ecosystem is good, it is far from the city and other conditions of the site are poor, causing the SA and HA coupling coordination index to be low.

#### 3.1.4. Coupling Coordination Relationship and Spatial Analysis of the PA-HA-SA

**Figure 7:**
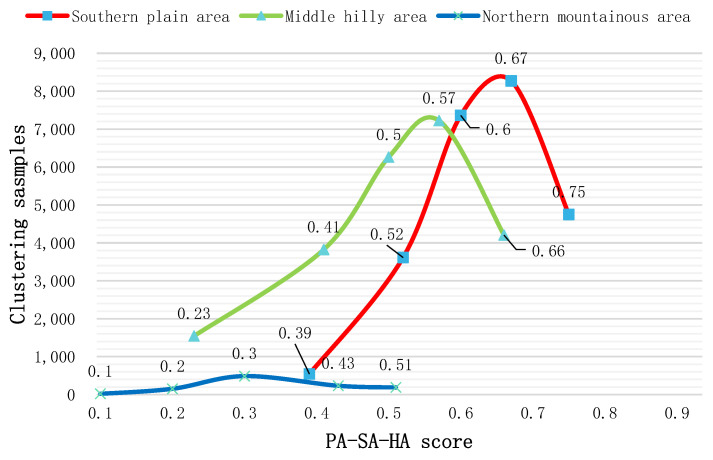
Clustering scores of the coupling coordination index of PA-HA-SA.

A comprehensive analysis of the coupling coordination relationship (Figure 3d) between PA-SA-HA shows that the mean coupling coordination index in the study area was 0.57, which is at the “barely coordination” level. This level describes a total of 31,805 parcels. In the NMA, the mean coupling coordination index was 0.34, which is at the “mild imbalance” level, and the amount of cultivated land is small. The K-means clustering method was used to analyze the cultivated land coupling coordination index in the MHA and SPA (Figure 7). In the MHA, the cluster center values were 0.23, 0.41, 0.50, 0.57, and 0.60, while the mean value was 0.51. In the SPA, the cluster centers were calculated as: 0.39, 0.52, 0.60, 0.67, and 0.75, with a mean value of 0.63, indicating that the relationship between PA, HA, and SA in this region is at the “primary coordination” level. In this sample, 83% of the parcels in this area are at the “primary coordination” level. We also found that the peak of cohesion in this region is relatively close, indicating that there was not a large difference between the coordination and coupling of cultivated land in this region. Compared with other regions, the peak value of the red curve index in this region is significantly higher, indicating that soil properties and geological conditions there are suitable for farming, heavy metal pollution is low in this region, and the site conditions of arable land are good. This spatial distribution is similar to the results of “Jiangyou City Cultivated Land Quality Grade Update Database”. The average National Utilization Grade of cultivated land in the MHA and SPA regions is about 7, which is far better than that in the NMA region [62]. The high level of coupling and coordination shows that the PA, HA, and SA subsystems in the study area can achieve benign interaction, common promotion, and coordinated development. Essentially, the productivity of the arable land can provide the necessary incentive for the development of the site conditions. In turn, the development of site conditions, such as road networks, irrigation canals, and more can also provide the necessary development conditions for increasing the productivity of arable land. At the same time, the development of arable land productivity, to a certain extent, encourages the government and farmers to control soil pollution, providing for the healthy development of arable land.

Based on our analysis, PA, HA, and SA subsystems of cultivated land have a relationship of mutual connection and influence as a result of the formation of an open and complex system. In the process of arable land use, all functions work together, and the disorderly development of any function will lead to the decline of the system’s coupling coordination degree. With the development of society, as the spatial carrier of urban and industrial development, the demand of human for construction land continues to increase, and the problems of “non-agricultural” and “non-food” of cultivated land continue to appear, which seriously affects the comprehensive function of cultivated land. At the same time, science and technology have made continuous progress. Pesticides and fertilizers, agricultural machinery, plastic films, etc., have been widely used in agricultural production, and the functions of agricultural production have been enhanced. However, the excessive use of pesticides and fertilizers will cause negative feedback to the cultivated land and affect the soil health. Therefore, the degree of coupling and coordination between different subsystems of cultivated land has an obvious impact on the comprehensive function of cultivated land. Analyzing the coupling and coordination of different systems of cultivated land can provide a scientific basis for improving the comprehensive function of cultivated land.

### 3.2. Application of the Coupling Coordination Degree System Based on PA-SA-HA

**Figure 8:**
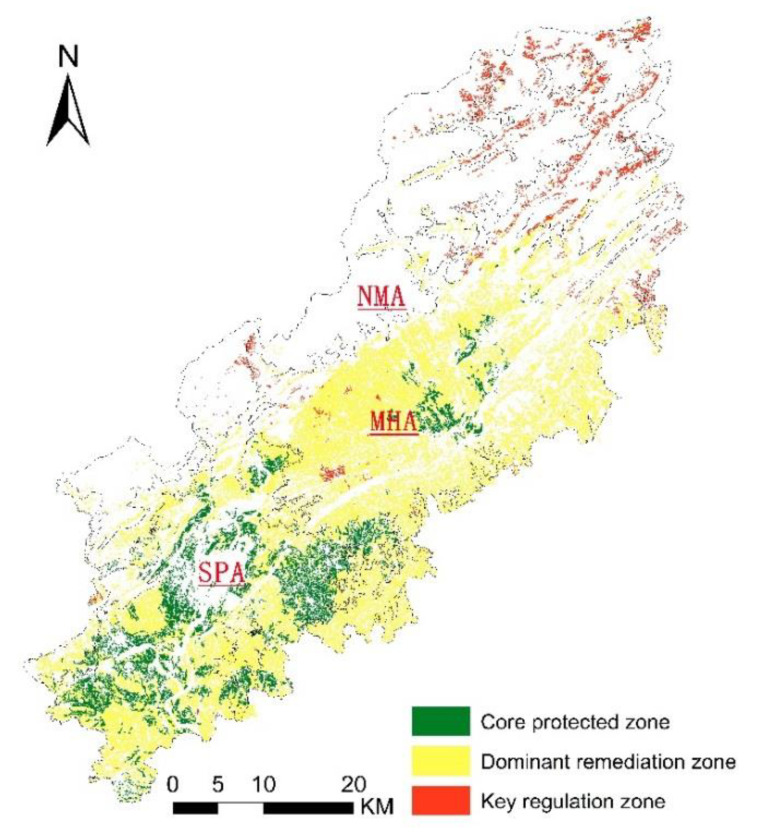
Cultivated land classification based on coupling coordination index.

Taking cultivated land as the evaluation unit, we classified the cultivated land parcels according to the coupling coordination index of PA-SA-HA. The index above the moderate coordination level is classified as core protected zone. The cultivated land whose index is located between the mild imbalance level and the moderate coordination level is classified as dominant maintenance zone. Additionally, the cultivated land with the index below the mild imbalance level is classified as a key regulation zone. As a result, we divided the cultivated land in the whole area into three types (Figure 8): the core protected zone of cultivated land resources, the dominant remediation zone, and the key regulation zone.

(1) The core protected zone of cultivated land resources

The area of cultivated land in this zone is 160.15 km^2^, which accounts for 19.32% of the cultivated land resources in Jiangyou City. The average coupling coordination indexes of PA-SA, PA-HA, SA-HA, and PA-SA-HA in this zone were: 0.83, 0.89, 0.78, and 0.72, respectively. The cultivated land resources in this area are mainly located in the southern plain of Jiangyou City, which is a river impact plain. There are many rivers, such as Fujiang River and Tongkou River, in this area. The zone has a flat terrain, fertile soil, complete irrigation facilities, and a high concentration of arable land. It has convenient location conditions and is appropriate for mechanized farming and modern agricultural development. The cultivated land is close to the water source, the soil is healthy, and it has good ecological and environmental characteristics. This area is the preferred area for high standard basic farmland construction and permanent basic demarcation.

(2) The dominant remediation zone of cultivated land resources

The total area of cultivated land in this zone is 611.25 km^2^, accounting for 73.72% of the cultivated land resources in the study area. The average coupling coordination index of PA-SA, PA-HA, SA-HA, and PA-SA-HA were calculated as: 0.65, 0.73, 0.60, and 0.56, respectively. The cultivated land resources in this area are mainly distributed in the MHA and the surrounding areas of Jiangyou City. The productivity of cultivated land in hilly areas is high, but the site conditions and the health of cultivated land are relatively poor. In particular, the areas surrounding Jiangyou City are far from the city center, the road network conditions are average, and the scale of cultivated land is small. In this zone, a small portion of the cultivated land resources in the SA plain have superior productivity, but the health conditions are poor. Therefore, these resources can require further improvement in the future.

In this zone, the cultivated land in the north of the MHA has great ecological functional value, but the land is fragmented. Compared with the core protection zone, the coupling coordination degree index of productivity and site conditions in this area is poor and there is room for improvement. Therefore, the zone should implement differentiated management and focus on protecting its advantages while improving its shortcomings. The site conditions of cultivated land in some areas should be improved by initiatives such as adding new roads. Some areas would benefit from better soil quality, which can be improved through methods such as phytoremediation, while others should be improved by land consolidation projects. If the differentiated management of the cultivated land is properly implemented, the sustainable development and long-term stable utilization of cultivated land resources in this zone will be realized.

(3) The key regulation zone of cultivated land resources

The total area of arable land in this area is 57.67 km^2^, which accounts for 6.96% of the cultivated land resources in Jiangyou City. The average coupling coordination indexes of PA-SA, PA-HA, SA-HA, and PA-SA-HA in this zone were calculated as: 0.42, 0.50, 0.37, and 0.27, respectively. The cultivated land in this area is limited by the terrain and planting conditions, such as the thin arable layer, the low content of organic matter, and the fragmented parcels. Therefore, the cultivated land in this area is less suitable for development and utilization. This area has neither good natural quality nor superior site conditions. There are many restrictions on the implementation of land projects, and it is difficult to reconstruct in this area. Therefore, one possible use for the cultivated land in this zone is implementing ecological engineering projects such as “returning cultivated land to forestry project” or “returning cultivated land to grassland project” [63]. It can also be used as an “land increase/decrease linked project” area to realize its unique land function value.

## 4. Conclusions

(1) The cultivated land area of the NMA, MHA, and SPA is 24.98 km^2^, 424.01 km^2^, and 380.85 km^2^, respectively. According to our analysis, the four average coupling coordination indexes in the NMA, MHA, and SPA are similar: SPA > MHA > NMA. This is due to the flat terrain in the south of the study area, the dense road network and irrigation canals, and the close proximity to the urban center. The overall quality of cultivated land is higher than the other two regions. The average coupling coordination index of PA-SA-HA in the study area is at the “barely coordination” level. In the SPA, the coupling coordination index of each subsystem is not significantly different. In this area, the productivity of cultivated land, site conditions and soil health form a virtuous circle, which can promote each other. It can be used as a high standard farmland construction and improvement area. In the MHA, the four indexes are all smaller than that of the SPA. This shows that there is room for improvement in the productivity, site conditions, and health of cultivated land. In the NMA, the overall coupling coordination of cultivated land is the worst. The productivity index of cultivated land is the lowest, which cannot be coordinated with other systems.
(2) Based on the results of the coupling coordination degree of cultivated land in different geomorphic areas, the whole of the cultivated land is divided into three types. The first type is the core protected zone of cultivated land resources, accounting for 19.30% of the land studied. In this zone, cultivated land productivity, site conditions, and cultivated land health characteristics have significant advantages; therefore, it can be used as the first choice for the delineation of high standard basic farmland and permanent basic farmland. The second type is the cultivated land dominant remediation zone, accounting for 73.66% of the total land studied. There is still room for improvement in the three subsystems of the cultivated land in this zone. Thus, although there are certain restrictions on the use of cultivated land here given its condition, comprehensive management of cultivated land resources can be implemented to alleviate the issues. In the area, the productivity of cultivated land can be improved by leveling the land, improving the soil, and increasing the investment in science and technology. The site conditions can be improved by merging scattered plots, building roads, expanding canals, and other measures. The health of cultivated land can be improved by building slope protection forests and controlling polluted land. For heavily polluted areas, farmland quality monitoring points shall be set up and continuously monitored to master the quality of farmland. The third type is the key regulation zone of cultivated land resources, accounting for 6.95% of the land studied. The natural quality and site conditions of arable land resources are relatively poor, there are many constraints on arable land resources, and it is difficult to renovate the arable land. In this zone, we advise implementing measures such as the ecological conversion of arable land, as well as “increase/decrease linked projects” to improve the effective utilization of the arable land resources. Under the premise of good ecological protection, the region can develop the under forest economy. It can also implement projects such as “closing mountains for afforestation” and “returning farmland to forests/grasslands” to improve ecological benefits.

However, this study has some limitations. For example, the soil evaluation index does not include indicators such as soil biota factor. The next step of the study will be to explore the specific application of these results in the delineation of “three zones and three lines”.

## Figures and Tables

**Table 1:** Dataset description and sources.

Evaluation System	Factors	Index	Data Source
PA	Geographical conditions	Land surface slope	Jiangyou City Land Use Status Database (2019)
	Soil properties	Available soil depth/cm	Jiangyou City Cultivated Land Quality Grade Update Database (2019)
		Soil texture	
		Soil organic matter/(g/kg)	
		Soil pH	
		Profile pattern	
SA	Location factors	Urban influence degree	Jiangyou City Land Use Status Database (2019)
		Influence degree of agricultural market	Baidu Map Open Platform (www.baidu.com)
	Farmland capital construction	patch shape index	Jiangyou City Land Use Status Database (2019)
		Water network density	
		Density of farmland road network	
	Social and economic factors	Cultivated land per capita	Jiangyou Yearbook 2019
HA	External HA	Fractional vegetation cover(FVC)	Geospatial Data Cloud (http://www.gscloud.cn, (accessed on 18 December 2020))
	Internal HA	Soil heavy metal	Jiangyou City Soil Database (2020)

**Table 2:** Comprehensive evaluation indicator system and quantification standards of cultivated land.

Evaluation System	Factors	Index	Classification Standard									
			100	90	80	70	60	50	40	30	20	10
PA	Geographical conditions	Land surface slope	<2°	2–5°	5–8°		8–15°			15–25°		≥25°
	Soil properties	Available soil depth	≥100	60–100			30–60					<30
		Soil texture	Loam soil		Clay soil		Sand soil		gravelly soil			
		Soil organic matter	≥40	30–40	20–30	10–20	6–10	<6				
		Soil pH	6.0–7.9	5.5–6.0/7.9–8.5	5.0–5.5/8.5–9.0		4.5–5.0				<4.5/>9.0	
		Profile pattern	Loam/loam/loam,loam/sand/loam; loam/sand/loam; loam/sand/loam	Loam/clay/loam		Sand/clay/sand,Loam/clay/clay,Loam/sand/sand	Sand/clay/clay	Clay/sand/clay,Clay/clay/clay,Clay/sand/sand	Sand/sand/sand,Gravel/gravel/gravel			
SA	Location factors	Urban influence degree	≥80	60–80			40–60				<40	
		Influence degree of agricultural market	≥70	50–70			30–70			<30		
	Farmland capital construction	Patch shape index	≥0.05		0.02–0.05		<0.02					
		Water network density	≥40		30–40		20–30		<20			
		Farmland road density	≥3		2–3		1–2		<1			
	Social and economic factors	Cultivated land per capita	≥0.3		0.2–0.3		0.1–0.2		<0.1			
HA	External HA	Fractional vegetation cover	>0.8		0.6–0.8		0.4–0.6		<0.4			
	Internal HA	Soil heavy metal	≤0.7		0.7–1.0		1.0–2.0		2.0–3.0		>3.0	

## Data Availability

Not applicable.
